# Extrapulmonary Tuberculosis (TB) Mimicking Meigs’ Syndrome: A Case Report

**DOI:** 10.7759/cureus.103727

**Published:** 2026-02-16

**Authors:** Nudrat Khan, Anika Ahmed, Esha Shiroley, Muhammad Mirza, Tinalo Molefe

**Affiliations:** 1 Acute Medicine, Peterborough City Hospital, Peterborough, GBR; 2 General Medicine, Peterborough City Hospital, Peterborough, GBR; 3 Cardiology, Peterborough City Hospital, Peterborough, GBR; 4 Respiratory Medicine, Peterborough City Hospital, Peterborough, GBR

**Keywords:** adnexal masses, anti-tb treatment, elevated ca-125, extrapulmonary tb, meigs´ syndrome, ovarian cyst, pseudo meig syndrome, tuberculous ascites, tuberculous pleural effusion

## Abstract

Meigs’ syndrome is characterized by the triad of a benign ovarian tumor, ascites, and pleural effusion, with resolution of fluid collections following tumor removal. However, a range of other pelvic pathologies can mimic this presentation, sometimes referred to as pseudo-Meigs’ syndrome. Extrapulmonary tuberculosis (EPTB) should be carefully considered as an important differential diagnosis in TB-endemic regions.

We report the case of a 34-year-old woman who presented with progressive abdominal distension, epigastric discomfort, and shortness of breath. Clinical examination and imaging revealed ascites, a large left-sided pleural effusion, and a complex adnexal mass, raising concerns for intra-abdominal malignancy or Meigs’ syndrome in the presence of elevated cancer antigen 125 (CA-125). Thoracoscopy with pleural biopsy was performed, which showed necrotizing granulomatous inflammation. Cytology of ascitic and pleural fluid demonstrated lymphocytic predominance without malignant cells, and microbiological cultures were negative. EPTB was diagnosed based on clinical, radiological, and histological findings. A nine-month course of standard anti-tubercular medications was started and demonstrated significant clinical recovery, including resolution of symptoms, weight gain, and radiological improvement.

This case illustrates how EPTB can closely mimic pseudo-Meigs’ syndrome, emphasizing the importance of considering TB in patients from endemic regions presenting with ascites, pleural effusion, and adnexal masses, even in the presence of raised tumor markers. Early biopsy and multidisciplinary evaluation are key to avoiding misdiagnosis and ensuring timely and effective treatment.

## Introduction

Extrapulmonary tuberculosis (EPTB) can present with diverse and often misleading clinical manifestations, particularly in endemic regions where the disease remains prevalent. EPTB accounts for approximately 15%-20% of all TB cases worldwide, with abdominal and peritoneal involvement representing a smaller but clinically significant proportion [1,2]. It may involve the peritoneum and pleura, producing features such as ascites, pleural effusion, and adnexal masses, findings that can closely mimic gynecologic malignancy. These manifestations may be radiologically indistinguishable from peritoneal carcinomatosis, and the paucibacillary nature of TB frequently precludes microbiological confirmation, making diagnosis reliant on clinical suspicion, imaging, and histopathological evaluation. 

A particularly challenging diagnostic scenario arises when EPTB mimics Meigs’ or pseudo-Meigs’ syndrome. Meigs’ syndrome, first described in the early 20th century, is classically defined by the triad of a benign ovarian tumor, ascites, and pleural effusion, with complete resolution of the effusions following tumor resection. A range of other pathological entities, including metastatic carcinoma, primary peritoneal tumors, and benign gynecologic conditions such as uterine fibroids and struma ovarii, can reproduce this constellation of findings, collectively referred to as pseudo-Meigs’ syndrome. Among these, EPTB represents an important, though often underrecognized, cause. Several case reports and small case series have described peritoneal or EPTB presenting with ascites, pleural effusion, adnexal masses, and elevated CA-125 levels, closely mimicking ovarian malignancy and Meigs' or pseudo-Meigs' syndrome [3, 4]. The overlap in clinical and radiologic features, compounded by elevated tumor markers such as CA-125, can further mislead clinicians toward a diagnosis of ovarian carcinoma. 

This case underscores the importance of maintaining a high index of suspicion for TB in women presenting with ascites, pleural effusion, and adnexal masses, especially in regions where the disease burden remains high. 

## Case presentation

A 34-year-old Indian woman presented with a four-week history of constant epigastric discomfort, progressive abdominal distension, and exertional shortness of breath associated with a dry cough. She denied weight loss, anorexia, or fever but reported fatigue. Her menstrual cycles were regular, and she had no significant past medical history. Before admission, she was treated in primary care with a proton pump inhibitor (omeprazole) and subsequently Helicobacter pylori eradication therapy for the above symptoms, without improvement. At this stage, she was further investigated for other causes of the above symptoms, which revealed a markedly elevated CA-125 (369 U/mL), prompting further investigations (Table 1).

**Table 1:** Initial investigations following admission. HCG, human chorionic gonadotropin

Test	Result	Reference value
White blood cells	3.7	4.0-11.0 x 10^9^/L
Red blood cells	4.34	3.8-5.8 x 10 ^12^/L
Hemoglobin	108	115-165 g/L
Platelets	345	150-400 x 10^9^/L
Mean corpuscular volume	77.4	80-100 fL
C-reactive protein	48	<5 mg/L
Albumin	35	35-50 g/L
Total bilirubin	4	0-21 umol/L
Alanine transaminase	13	<33 IU/L
Alkaline phosphatase	51	30-130 IU/L
Total protein	74	60-80 g/L
Sodium	136	133-146 mmol/L
Potassium	4.2	3.5-5.3 mmol/L
Creatinine	35	45-84 mmol/L
Urea	1.4	2.5-7.8 mmol/L
Estimated glomerular filtration rate	>90	>60 mL/min/1.73 m^2^
Carbohydrate antigen 125	369	<35 U/mL
Alpha fetoprotein	<2	<7 ng/mL
Carbohydrate antigen 19-9	12	<27 U/mL
Carcinoembryonic antigen	<1	<5 ug/L
Serum HCG	<1	<5 U/L
Lactate dehydrogenase	203	<250 IU/L

**Figure 1:**
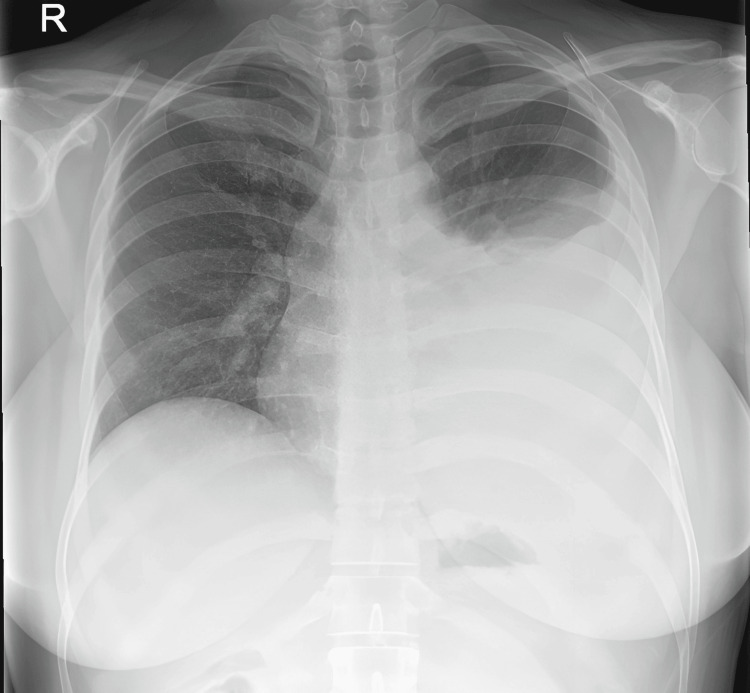
Initial chest radiograph (CXR) demonstrating a left-sided pleural effusion.

On admission, she was otherwise hemodynamically stable except for tachycardia. Examination revealed reduced air entry at the left lung base and a distended abdomen with epigastric tenderness. Laboratory investigations demonstrated a mild microcytic anemia (hemoglobin 108 g/L, mean corpuscular volume 77 fL) and raised C-reactive protein (48 mg/L), while other tumor markers were within normal limits. Chest radiography showed a large left pleural effusion (Figure 1).

**Figure 2:**
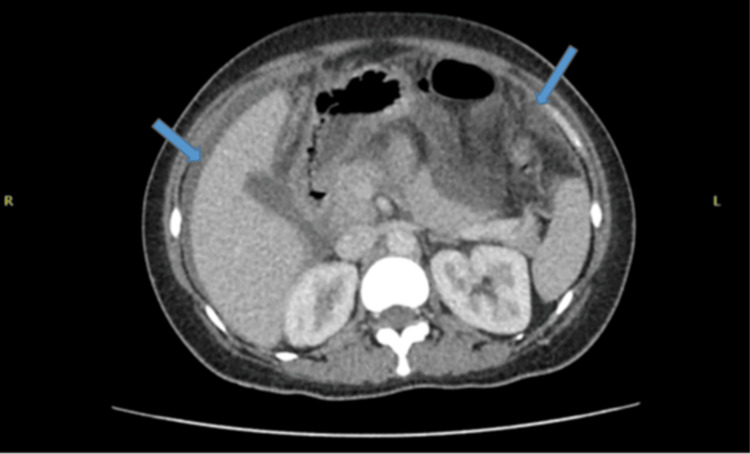
Cross-sectional CT images showing ascites and peritoneal thickening (blue arrows).

**Figure 3:**
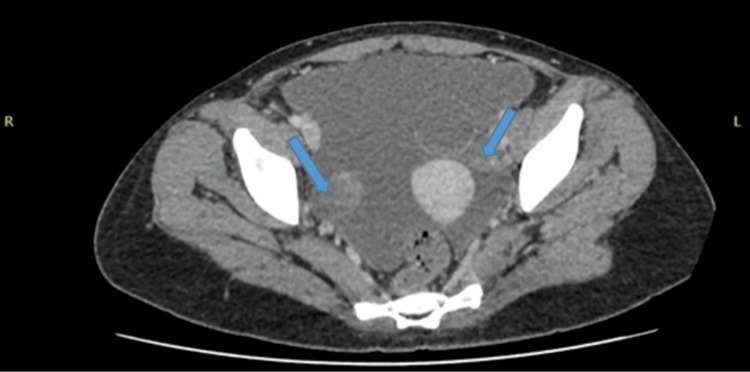
Cross-sectional CT image showing bilateral adnexal changes (blue arrows).

**Figure 4:**
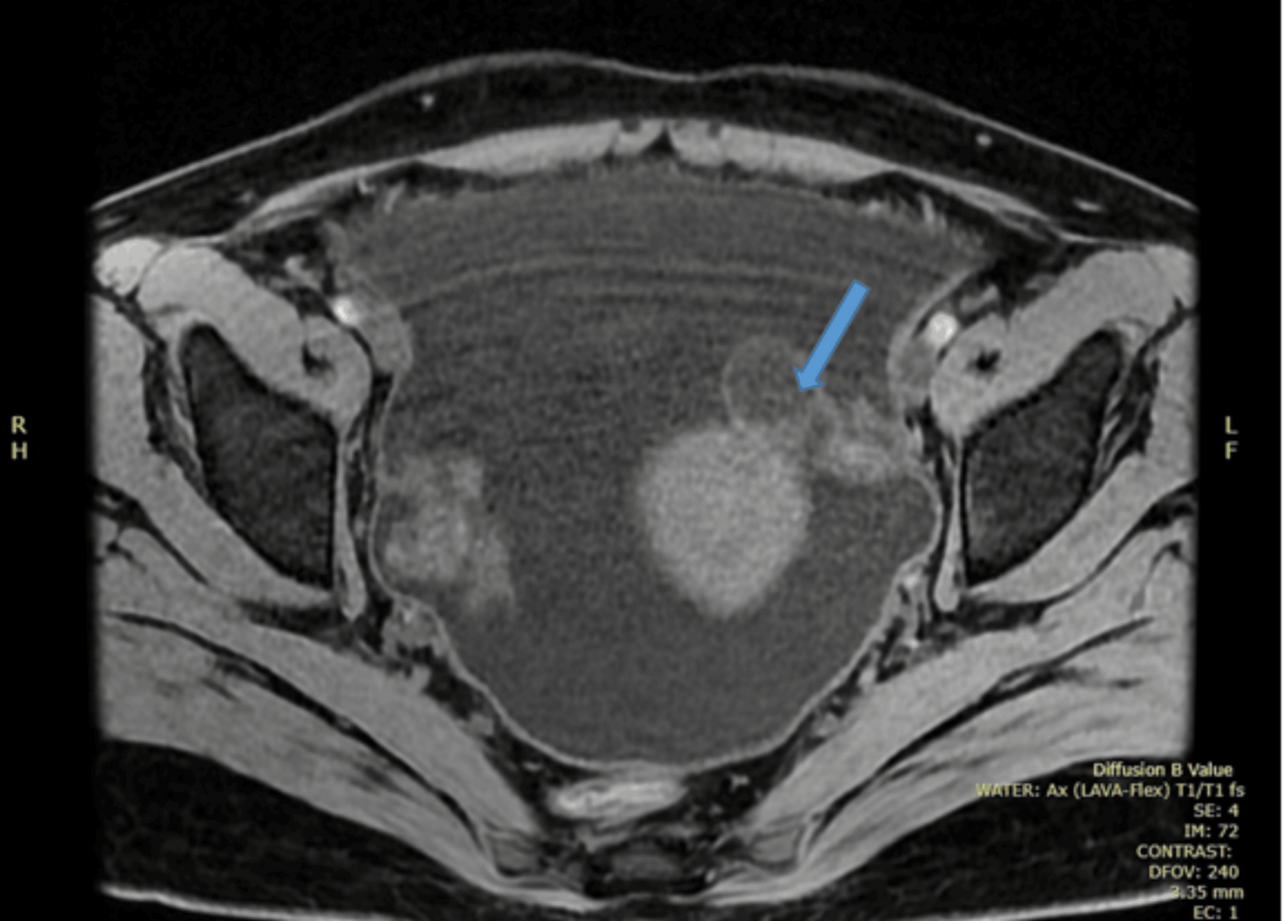
Initial MRI demonstrating a left ovarian cyst (blue arrow). MRI, magnetic resonance imaging

An abdominal ultrasound confirmed gross ascites. Cross-sectional imaging (computed tomography (CT) chest/abdomen/pelvis and MRI pelvis) demonstrated a large pleural effusion, extensive ascites, peritoneal thickening, and bilateral adnexal changes with a complex left ovarian cyst (Figures 2-4).

In view of the raised CA-125 and these findings, the initial differential diagnoses included peritoneal carcinomatosis or Meigs’ syndrome. The case was discussed at the gynecology multidisciplinary meeting, which advised tissue diagnosis. The patient was referred to the respiratory team as well and subsequently underwent medical thoracoscopy for tissue diagnosis and symptomatic relief, draining 2.6 L of hemorrhagic fluid. Cytological examination of pleural and ascitic fluid revealed a lymphocytic predominance without malignant cells. Pleural histology demonstrated extensive necrotizing granulomatous inflammation with multinucleated giant cells, consistent with tuberculosis, and no evidence of malignancy. Microbiological cultures, including acid-fast bacilli smear, culture, and PCR, were negative on pleural fluid and pleural biopsy samples. ADA levels and GeneXpert MTB/RIF testing were not performed, as the main focus was on ruling out malignancy due to raised CA-125 and imaging findings (Tables 2-3).

**Table 2:** Pleural fluid report.

Test	Result	Typical reference range/interpretation
Fluid protein	57 g/L	Transudate: <25 g/L; exudate: >35 g/L
Fluid LDH	279 IU/L	Transudate: <200 IU/L; exudate: >200 IU/L or >2/3 upper limit of normal serum LDH
White blood cells	+	Normally <1,000 cells/uL
Red blood cells	++	Normally very low, typically <10,000/uL, suggesting traumatic tap or malignancy
Organisms (microscopy)	Bacteria not seen	Normal: No organisms
Culture	No growth	Normal: No growth

**Table 3:** Ascitic fluid report. HTN, hypertension; PMNs, polymorphonuclear leukocytes; SAAG, serum-ascites albumin gradient

Test	Result	Typical reference range/interpretation
Fluid albumin	28 g/L	Ascitic fluid albumin varies; used to calculate SAAG; typical: 714 g/L in non-portal HTN; higher in malignant ascites
Fluid glucose	4.5 mmol/L	3.35-5.5 mmol/L
White blood cells	1.917 × 10^9^/L	Normal <0.5 × 10^9^/L
Polymorph cells (PMN count)	0.268 × 10^9^/L	Normal <0.25 × 10^9^/L

The clinical, radiological, and histological findings were consistent with extrapulmonary tuberculosis presenting as a pseudo-Meigs’ syndrome, and a diagnosis of extrapulmonary tuberculosis involving the pleura, peritoneum, and adnexa was established.

Standard anti-tubercular therapy was initiated according to the WHO consolidated tuberculosis treatment guidelines. The standard regimen for drug-susceptible tuberculosis, including most forms of EPTB, is two months of isoniazid (5 mg/kg/day), rifampicin (10 mg/kg/day), pyrazinamide (25 mg/kg/day), and ethambutol (15 mg/kg/day) during the intensive phase, followed by four months of isoniazid and rifampicin in the continuation phase. In our patient, pyrazinamide was withheld due to a history of gout, and ethambutol was continued into the continuation phase. The total duration of therapy was nine months (intensive phase: two months; continuation phase: seven months) to ensure adequate treatment response, given the regimen modification. Pyridoxine supplementation was provided throughout therapy.

**Figure 5:**
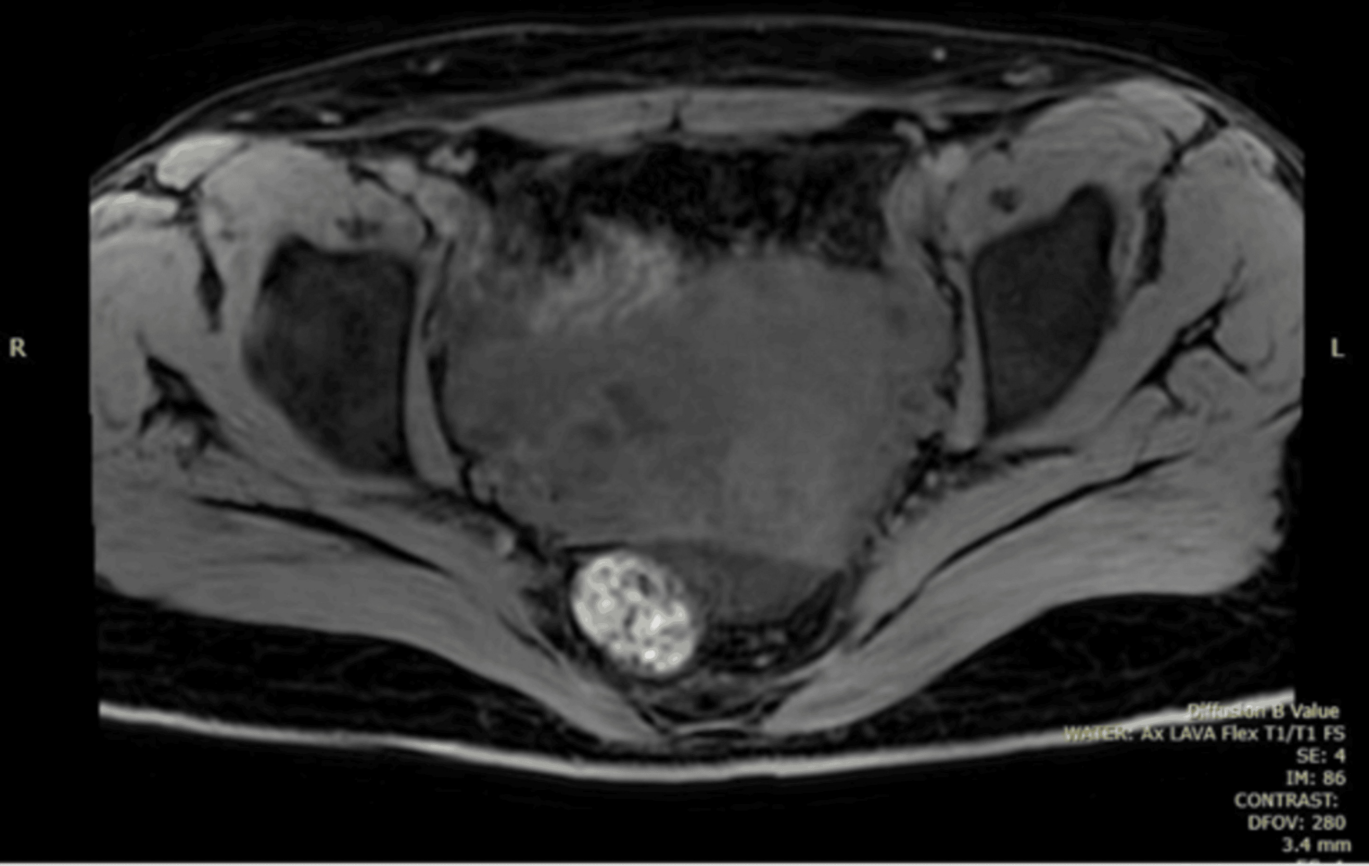
Repeat imaging showing resolution of adnexal changes.

On follow-up, the patient demonstrated marked clinical improvement, with resolution of abdominal pain, breathlessness, and cough, accompanied by weight gain. Repeat MRI pelvis at three months showed near-complete resolution of ascites and adnexal changes (Figure 5).

The indwelling pleural catheter was removed, and subsequent follow-up over the following year confirmed sustained recovery with no evidence of malignancy.

## Discussion

This case illustrates EPTB presenting with ascites, pleural effusion, and an adnexal mass - features that closely mimic Meigs’ or pseudo-Meigs’ syndrome. Such presentations pose significant diagnostic challenges, as elevated CA-125 levels and imaging findings may initially suggest ovarian malignancy. 

EPTB represents approximately 15% of all TB infections, most commonly affecting the lymphatic system. In women, the peritoneum, pleura, and genitourinary tract may also be involved, occasionally mimicking gynecological malignancy [3]. Traditionally, the main reported risk factors associated with the development of EPTB include extremes of age (<15 and > 65 years of age), female sex, migration from high-incidence TB regions, and immunosuppression [5]. Globally, 7.5 million new or relapsed cases of TB were notified in 2022, of which 83% were pulmonary, and 17% represented EPTB [1]. 

The pathophysiology of EPTB differs depending on the organ system affected. Nevertheless, granuloma formation following reactivation and hematogenous spread is a shared feature across different forms of EPTB [6].

EPTB, particularly in women, can present with symptoms such as abdominal pain, bloating, ascites, and elevated CA-125 levels-clinical features that may closely resemble Meigs’ syndrome or pseudo-Meigs’ syndrome, which are typically associated with an ovarian tumor, ascites, and pleural effusion [4]. This similarity can lead to a misdiagnosis of malignancy. Abdominal TB may present with an abdominal mass and fluid accumulation, mimicking the presentation of gynecologic cancers. The role of CA-125 in EPTB remains uncertain, as most of the data come from case reports or small series, making it difficult to establish a reliable correlation between elevated CA-125 levels and the presence of EPTB. It is hypothesized that elevated CA-125 reflects peritoneal or pleural inflammation, as CA-125 is expressed by mesothelial cells lining these surfaces [4]. In our patient, elevated CA-125 contributed to initial concern for ovarian malignancy, highlighting the diagnostic challenge.

The diagnosis of EPTB relies on a combination of clinical suspicion and multiple diagnostic tools, as no single test is definitive. Mycobacterial stain and culture remain the gold standard but have low sensitivity in EPTB due to the paucibacillary nature of specimens and the slow turnaround time of up to eight weeks [7]. Biopsy of affected tissues, often guided by imaging or endoscopy, offers higher diagnostic accuracy, particularly when histopathologic findings (granulomas, caseation) are combined with culture and polymerase chain reaction (PCR) testing [8]. Body fluid analysis (pleural, peritoneal, cerebrospinal fluid (CSF), pericardial) can provide supportive evidence, with adenosine deaminase (ADA) levels being a widely used marker because of their high sensitivity and specificity at certain cutoffs, although false results are possible [9]. Nucleic acid amplification tests (NAATs), particularly the Xpert MTB/RIF assay, enable rapid detection of TB and rifampicin resistance, with sensitivity varying by specimen site, high in lymph nodes and CSF, and lower in pleural fluid [10]. In this case, ADA levels and GeneXpert MTB/RIF testing were not performed, as the initial clinical focus was on ruling out malignancy due to raised CA-125 and imaging findings.  Immunological tests, including the tuberculin skin test (TST) and interferon-gamma release assays (IGRAs), are of limited diagnostic value in EPTB, as they cannot distinguish latent from active disease and perform variably across different disease sites. Overall, an integrated approach using microbiology, histology, molecular tests, and fluid biomarkers-tailored to the site of infection-is essential for accurate and timely diagnosis of EPTB. 

Anti-TB drug therapy remains the mainstay of EPTB management. Although treatment regimens remain controversial due to limited high-quality evidence specific to EPTB, most guidelines recommend using the same regimen as for pulmonary TB (PTB), typically a six-month course, but adjustments are needed based on the specific site of disease involvement [11]. Monitoring treatment response is challenging due to difficulties in obtaining follow-up specimens. Unlike PTB, bacteriological confirmation of cure is often not feasible; thus, clinicians primarily rely on clinical and radiographic improvements, which vary by disease site and severity. There are currently no standardized criteria for defining cure or treatment completion in EPTB [2].

## Conclusions

This case demonstrates EPTB to closely mimic Meigs’ syndrome, thereby posing a considerable diagnostic challenge. The triad of ascites, pleural effusion, and an adnexal mass, when combined with elevated CA-125 levels, almost invariably raises concern for ovarian malignancy, often prompting invasive procedures or surgical intervention. However, as this report illustrates, TB can produce an identical clinical and radiological picture, making it imperative for clinicians to maintain a high index of suspicion, especially in patients from endemic regions or those with epidemiological risk factors.

A multidisciplinary approach and histological confirmation via pleural or peritoneal biopsy remain essential, as fluid cytology, microbiological cultures, and nucleic acid amplification tests (Xpert MTB/RIF) may often be inconclusive in EPTB, particularly in paucibacillary samples, as in our patient. Early tissue diagnosis not only prevents unnecessary surgery but also allows for the initiation of anti-tubercular therapy, which can achieve complete clinical and radiological resolution. Prompt recognition and appropriate management can lead to excellent outcomes, reduce healthcare costs, and prevent the morbidity associated with delayed or incorrect treatment pathways.
